# Combined Analysis of RNA Sequence and Microarray Data Reveals a Competing Endogenous RNA Network as Novel Prognostic Markers in Malignant Pleural Mesothelioma

**DOI:** 10.3389/fonc.2021.615234

**Published:** 2021-04-23

**Authors:** Weicheng Duan, Kang Wang, Yijie Duan, Xiuyi Chen, Xufeng Chu, Ping Hu, Bo Xiong

**Affiliations:** ^1^Department of Forensic Medicine, Tongji Medical College, Huazhong University of Science and Technology, Wuhan, China; ^2^Key Laboratory of Environment and Health (HUST), Ministry of Education, School of Public Health, Tongji Medical College, Huazhong University of Science and Technology, Wuhan, China

**Keywords:** ceRNA, mesothelioma, lncRNA, biomarker, overall survival, microenvironment

## Abstract

Malignant pleural mesothelioma (MPM) is a highly aggressive cancer with short survival time. Unbalanced competing endogenous RNAs (ceRNAs) have been shown to participate in the tumor pathogenesis and served as biomarkers for the clinical prognosis. However, the comprehensive analyses of the ceRNA network in the prognosis of MPM are still rarely reported. In this study, we obtained the transcriptome data of the MPM and the normal samples from TCGA, EGA, and GEO databases and identified the differentially expressed (DE) mRNAs, lncRNAs, and miRNAs. The functions of the prognostic genes and the overlapped DEmRNAs were further annotated by the multiple enrichment analyses. Then, the targeting relationships among lncRNA–miRNA and miRNA–mRNA were predicted and calculated, and a prognostic ceRNA regulatory network was established. We included the prognostic 73 mRNAs and 13 miRNAs and 26 lncRNAs into the ceRNA network. Moreover, 33 mRNAs, three miRNAs, and seven lncRNAs were finally associated with prognosis, and a model including seven mRNAs, two lincRNAs, and some clinical factors was finally established and validated by two independent cohorts, where CDK6 and SGMS1-AS1 were significant to be independent prognostic factors. In addition, the identified co-expressed modules associated with the prognosis were overrepresented in the ceRNA network. Multiple enrichment analyses showed the important roles of the extracellular matrix components and cell division dysfunction in the invasion of MPM potentially. In summary, the prognostic ceRNA network of MPM was established and analyzed for the first time and these findings shed light on the function of ceRNAs and revealed the potential prognostic and therapeutic biomarkers of MPM.

## Introduction

Malignant pleural mesothelioma (MPM), which is mainly associated with the asbestos exposure and derived from the pleural or peritoneal mesothelial cell surfaces, is an aggressive tumor with very poor prognosis (median survival time <12 months after diagnosis) (1). According to epidemiological investigations, the number of patients has been increasing in recent years, especially in developing countries, which leads to ca. 40,000 deaths per year worldwide (2). Currently, it is difficult to differentially diagnose MPM, since inclusion of more biomarkers based on the latest research would likely increase the accuracy and efficiency of prognosis. It is therefore essential and urgent to identify new prognostic biomarkers and therapeutic targets and to understand its molecular mechanisms.

The competing endogenous RNA (ceRNA) theory illustrates a novel regulatory mechanism for gene regulation, by which one transcript (e.g., an lncRNA) can control and modulate the expression of another transcript (e.g., an mRNA) by competitive interactions with an miRNA (3). It has been demonstrated that ceRNAs are widely involved in the occurrence and progression of various cancers (4–6). Moreover, some ceRNAs have been shown to be valuable targets for the treatment and prognosis of multiple cancers (7, 8). With the development of high-throughput sequencing technologies, more and more ceRNA networks have been constructed and analyzed in multiple cancers, such as gastric cancer and breast cancer (9, 10). However, there are few studies focused on the function of ceRNA networks in MPM. Some recent studies implicated that non-coding RNA (ncRNA) dysfunction is closely associated with the properties of MPM cells (11–13). Therefore, it is highly likely that certain ceRNA networks may also be involved in the pathogenesis of MPM.

In this study, we analyzed the differential expression of mRNAs, lncRNAs, and miRNAs (DEmRNAs, DElncRNAs, and DEmiRNAs) based on the RNA-Seq and miRNA-Seq data in different subgroups of the MPM patients categorized according to the overall survival of the MPM patients. The DEGs related to the overall survival were further compared with the DEGs between the MPM and the normal tissues in the microarray data. Then, we constructed a ceRNA network of the overlapped DERNAs based on their interactions obtained from multiple databases. Furthermore, Kaplan–Meier survival analyses and univariate, lasso, and multivariate Cox regression analyses were conducted to explore the mRNA, miRNA, and lncRNA biomarkers in the ceRNA network. Accordingly, the risk assessment model combined with the multiple clinical factors and the screened RNAs based on regression coefficients were established, evaluated, and validated based on the two independent MPM datasets. Weighted gene co-expression network analysis (WGCNA) was used to further explore the reliability of the ceRNA network as a prognostic marker as well as its potential mechanism. This study provides new insights on the biological functions related to the lncRNA in MPM patients and the additional biomarkers for the prognosis of MPM.

## Materials and Methods

### TCGA MPM Dataset

Data for patients with MPM collected from the TCGA database was regarded as a training group. The criteria of exclusion were set as follows: (1) patients without lncRNA and mRNA information; (2) survival time of patients was unavailable or survival time of alive patients was <30 months. Overall, 80 MPM patients were included in our study. Next, the 80 patients were further screened to be included into two groups according to the survival time: high-risk group and low-risk group. The criteria of grouping were set as follows: patients with overall survival <12 months were included into high-risk group, and patients with overall survival >30 months were included in the low-risk group. Finally, the expression profiles of the patients in the two groups were used for the differential expression gene (DEG) analyses. In addition, the clinical information and the expression profiles of all 80 patients were used for the univariate, the lasso and multivariate Cox regression analyses, and WGCNA. Similarly, another study from the EGA database including 211 MPM patients was used to validate the RNA expression results. The GSE12345 (four normal tissues and nine MPM tissues) and GSE42977 (nine normal tissues and 39 MPM tissues) datasets were downloaded from the GEO database to explore the DEGs between the normal tissues and the MPM tissues for cross comparison of DEG associated with overall survival.

### Data Processing

The normalized read-count data and the Fragments Per Kilobase of transcript per Million fragments of RNA-Seq by RNASeqV2 were obtained from the TCGA database as well as the STAD level 3 microRNA sequencing (miRNA-seq) data. The cleaned fastq files were downloaded from the EGA database. According to the protocol in the TCGA database, the STAR software was used to align the fastq files to the human GRCh38 genome file, and the Htseq-Count software was used to count the reads. Next, the Edge R package was applied to analyze the gene expression profiles to identify the DEmRNAs, DElincRNAs, and DEmiRNAs (log_2_-fold change > 1.0, false discovery rate (FDR) < 0.05). The TPM (Transcripts Per Million) were calculated for the subsequent analyses and validation. The GSE12345 dataset obtained from the Affymetrix Human Genome U133 Plus 2.0 Array was annotated by GPL570. The GSE42977 dataset based on the Illumina HumanRef-6 v2.0 expression beadchip was annotated by GPL6790. The two microarray datasets were normalized by the robust multi-array average algorithm. When multiple probes were mapped to the same gene, the mean of the probe intensities will be used. After that, the differential expression analyses of the microarray data were performed using limma package [fold change > 2.0, false discovery rate (FDR) < 0.05].

### Functional Enrichment Analysis

The functional enrichment analyses of the DEGs, including GO function analyses and KEGG pathway analyses, were carried out by the clusterProfiler package. For the GO analyses, cellular component (CC), biological process (BP), and molecular function (MF) terms were analyzed. FDR < 0.05 was used as the statistically significant cutoff in these analyses. Besides, we also performed the gene set enrichment analysis (GSEA) for all the mRNAs using the clusterProfiler package with the c2.all.v6.2.entrez.gmt and c5.all.v6.2.entrez.gmt gene set collections. Gene sets with FDR < 0.05 were considered to be significantly enriched.

### Construction of an lncRNA-Related ceRNA Network

The overlapped DEGs between the RNA-Seq and the microarray were used for the construction of the prognostic ceRNA network. We used starBase v3.0 (http://starbase.sysu.edu.cn/) to retrieve the lncRNA–miRNA interactions and then used miRBase targets (http://mirdb.org/miRDB/), miRTarBase (http://mirtarbase.mbc.nctu.edu.tw/), and TargetScan (http://www.targetscan.org/) to predict lmiRNA–mRNA interactions (14–17). To increase the reliability of the results, only the miRNA–mRNA interactions intersecting in all three databases were included into the ceRNA network. Moreover, the Pearson correlations of the pairs between miRNAs and mRNAs were calculated and the absolute values of the pairs more than 0.4 were regarded as interacted pairs. Finally, a ceRNA network was constructed and visualized by Cytoscape v3.7.1.

### Survival Prediction Model Construction and Evaluation

The mRNAs, miRNAs, and lncRNAs from the ceRNA network were used for the univariable Cox regression analyses to screen for the RNAs associated with overall survival, and only the RNAs with *P* < 0.05 were enrolled into the followed Kaplan–Meier survival analyses based on the log-rank test according to the grouping by the median value of the expression quantities. After Kaplan–Meier survival analyses, the mRNAs and lncRNAs with *P* < 0.05 were included into the lasso Cox regression analyses to further screen the variables. Accordingly, the survival prediction model was constructed through the regression coefficients of the multivariable Cox regression analysis, as described in the following equation: risk score = R1G1 + R2G2 + R3G3 + ……RnGn (R is the regression coefficient obtained from the final multivariable Cox analysis and L is the expression value of each gene or the type of different clinical factor). Then, the patients were divided into two groups (high-risk-score group and low-risk-score group) according to the cutoff value obtained from the OptimalCutpoints package, and the Kaplan–Meier survival analyses were performed between the two groups. Moreover, the receiver-operating characteristic (ROC) curve analyses were performed to estimate the efficiencies of the model under the same grouping; only AUC values > 70% were regarded as efficient models.

### Identification and Annotation of Gene Modules Related to the Risk Model According to WGCNA

The WGCNA algorithm was used to construct the gene co-expression network (18). We first conducted the WGCNA analysis to identify the gene modules closely related to the overall survival and the risk scores. Then the significant modules were further selected for further analyses, and the mRNAs of the ceRNA network co-expressed in the significant modules were further input in the String database (string.org) to predict the protein–protein interaction (PPI) relationship. The PPI network was then visualized by Cytoscape v3.7.1, and the hub genes were identified using 12 algorithms of the CytoHubba plugin. Then, the results were cross-compared to generate a final list of hub genes. Besides, the genes enrolled into these significant modules were used for GO-BP, GO-MF, and KEGG pathway enrichment analyses by clusterProfiler package.

### Evaluation of the Tumor Infiltrating Immune Cells With the Risk Model

To infer the infiltrating immune cells of MPM associated with the ceRNA axes, we used the mRNAs in the ceRNA network to estimate the proportions of the 22 types of infiltrating immune cells by deconvolution of the CIBERSORT algorithm (19). According to the grouping, we obtained the significant changes of the immune cell types between the high- and low-risk-score groups using Student's *t*-test.

### Statistical Analysis

The differential expression gene analyses of the RNA-seq data were performed by the R package “edge R.” The differential expression gene analyses of the microarray data were conducted by the R package “limma.” The enrichment analyses were performed by R package “clusterProfiling.” All of these analyses involved in the multiple-hypothesis test were finally corrected by the FDR method. The univariate and the multivariate Cox regression analyses were performed by R package “survival.” The lasso regression analyses were performed by the R package “glmet.” Moreover, the Kaplan–Meier analyses were carried out by R package “survival,” whose statistical significances were assessed by the two-sided log-rank test. The statistical analyses between the clinical traits and the risk model were performed by the chi-square test or Fisher exact test according to the distribution of the data. The R software version 3.6.3 was used for the study. All statistical tests were two-sided.

## Results

### Screening of Patients and Identification of DEGs

**Figure 1:**
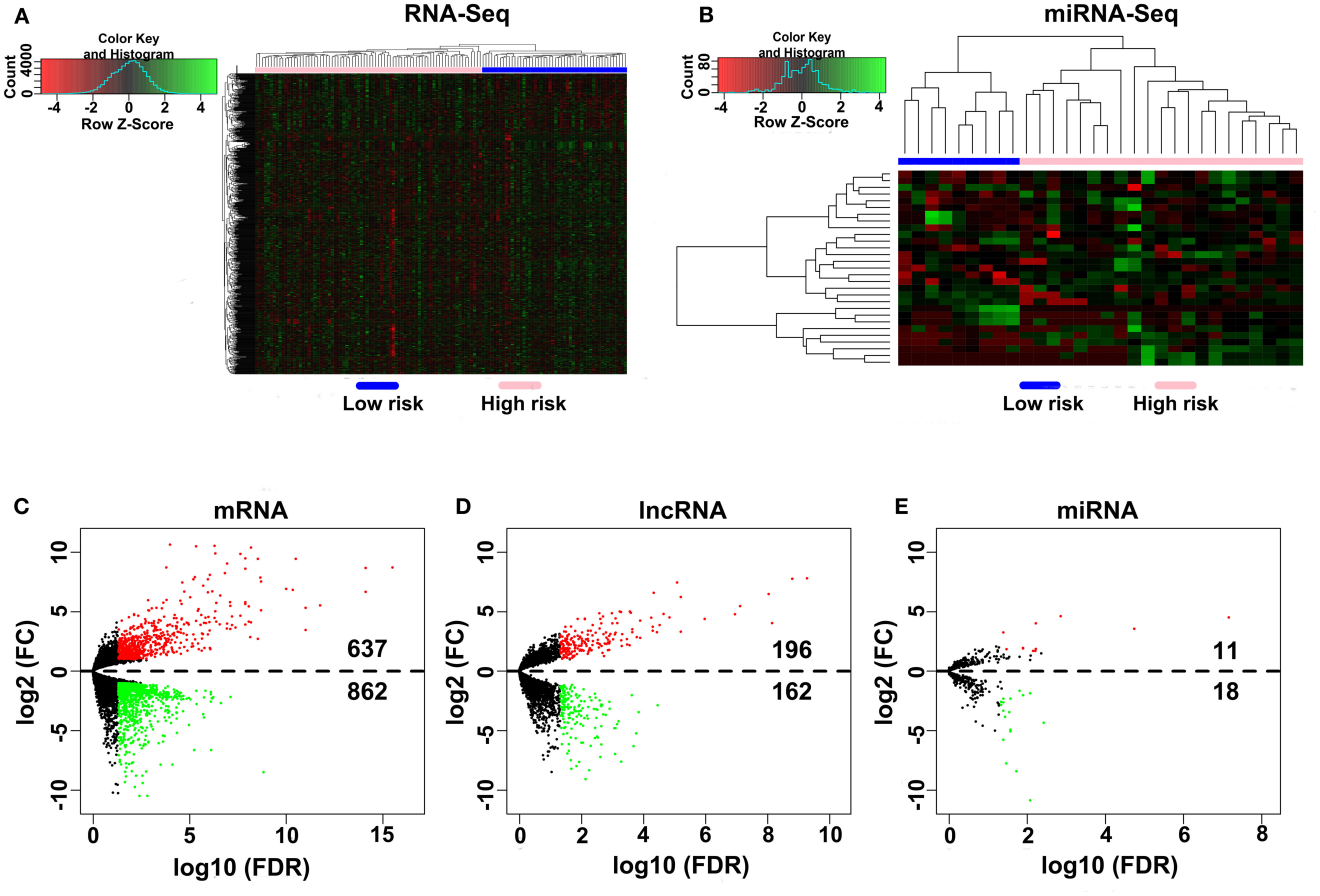
Differential expression gene analyses. **(A,B)** DEG heatmap of the RNA-Seq and the miRNA-Seq. **(C–E)** Screening of the DEmRNA, DElncRNA, and DEmiRNA. Black points represent the insignificant genes, red points represent the upregulated genes, and green points represent the downregulated genes. FC, fold change. Threshold criteria: FC > 2 and FDR < 0.05.

A total of 80 patients with MPM were downloaded from the TCGA database. Thereinto, the RNA expression profiles of 21 low-risk (overall survival > 30 months) samples and nine high-risk (overall survival < 12 months) samples were used to explore the DEmRNAs, DElncRNAs, and DEmiRNAs. The clinical and pathological characteristics of MPM patients are shown in Table 1. According to the results of the DEGs, the patients were clearly clustered into two groups, indicating that the overall gene expression patterns are significantly different in the two groups (Figures 1A,B). A total of 1,499 DEmRNAs, 358 DElncRNAs, and 29 DEmiRNAs were obtained according to the criteria (*P* < 0.05 and fold change > 2, Figures 1C–E).

**Table 1:** The characteristics of the included MPM patients.

**Characteristic**		**TCGA**		**EGA**	
		**All case numbers**	**Case numbers used in DEG analysis**	**All case numbers**	**Case numbers used in DEG analysis**
Sample type	MESO	80	30	211	128
Age	Median	64.5	62.5	65.4	64.15
	Range [years]	28–81	28–81	18.8–86	27.3–86
Sex	Male	65	27	176	108
	Female	15	3	35	20
Vital status	Alive	8	0	48	0
	Dead	72	30	163	128
Survival time	Median	15.06	8.32	12.6	7.8
	Range [months]	0.65–91.73	0.65–91.73	0.24–132.6	0.24–132.6

**Figure 2:**
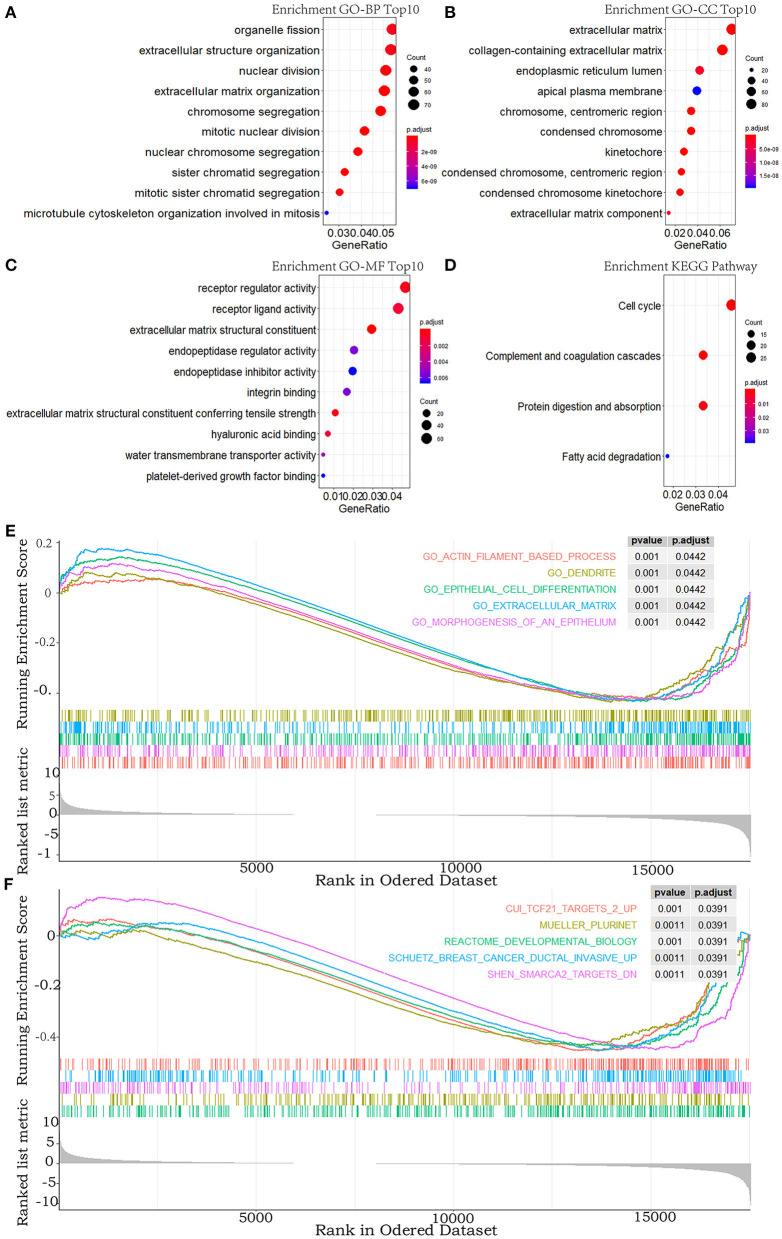
GO, KEGG pathway, and GSEA enrichment analyses. **(A–C)** Top 10 enriched terms in GO-BP, GO-CC, and GO-MF. **(D)** The significant terms in the KEGG pathway. **(E,F)** TOP 5 enriched terms of GSEA in the c5.all.v6.2.entrez.gmt gene sets and the c2.all.v6.2.entrez.gmt gene sets. *P-*value adjusted by FDR < 0.05.

To explore the functional features of these DEGs related to overall survival, we performed GO and KEGG enrichment analyses (FDR-corrected *P* < 0.05). The top 10 significant GO terms are mainly involved in the processes and the components related to the extracellular matrix, the cell division, and the receptor regulator activity (Figures 2A–C). Four pathways were enriched in the KEGG analysis (Figure 2D). These results all suggested that abnormal extracellular matrix components, cell cycle, and immune-response components are potentially important causes in the progression of MPM, which have been closely associated with growth and metastasis of other cancer cells (20).

In addition, to explore the changes of the overall gene expression levels of MPM, 17746 mRNAs were input to implement GSEA based on the fold change order using the gene sets of c2.all.v6.2.entrez.gmt and c5.all.v6.2.entrez.gmt. One hundred eighty terms in c5.all.v6.2.entrez.gmt gene set and 188 terms in the c2.all.v6.2.entrez.gmt gene set were enriched (FDR < 0.05), the top five of which were exhibited in Figures 2E,F. Morphogenesis and differentiation of epithelium, cell movement, and extracellular matrix were significantly enriched in the c5.all.v6.2.entrez.gmt gene set (Figure 2E), supporting the role of the extracellular matrix in the progression of MPM and indicating the convergence between the invasion of MPM and the morphogenesis and differentiation of the epithelium. Some specific targets and pathways were mainly enriched in the c2.all.v6.2.entrez.gmt gene set (Figure 2F), suggesting that progression of MPM may partly be involved in the invasive pathways of breast cancer and pathways related to TCF21 and SMARCA2 targets.

**Figure 3:**
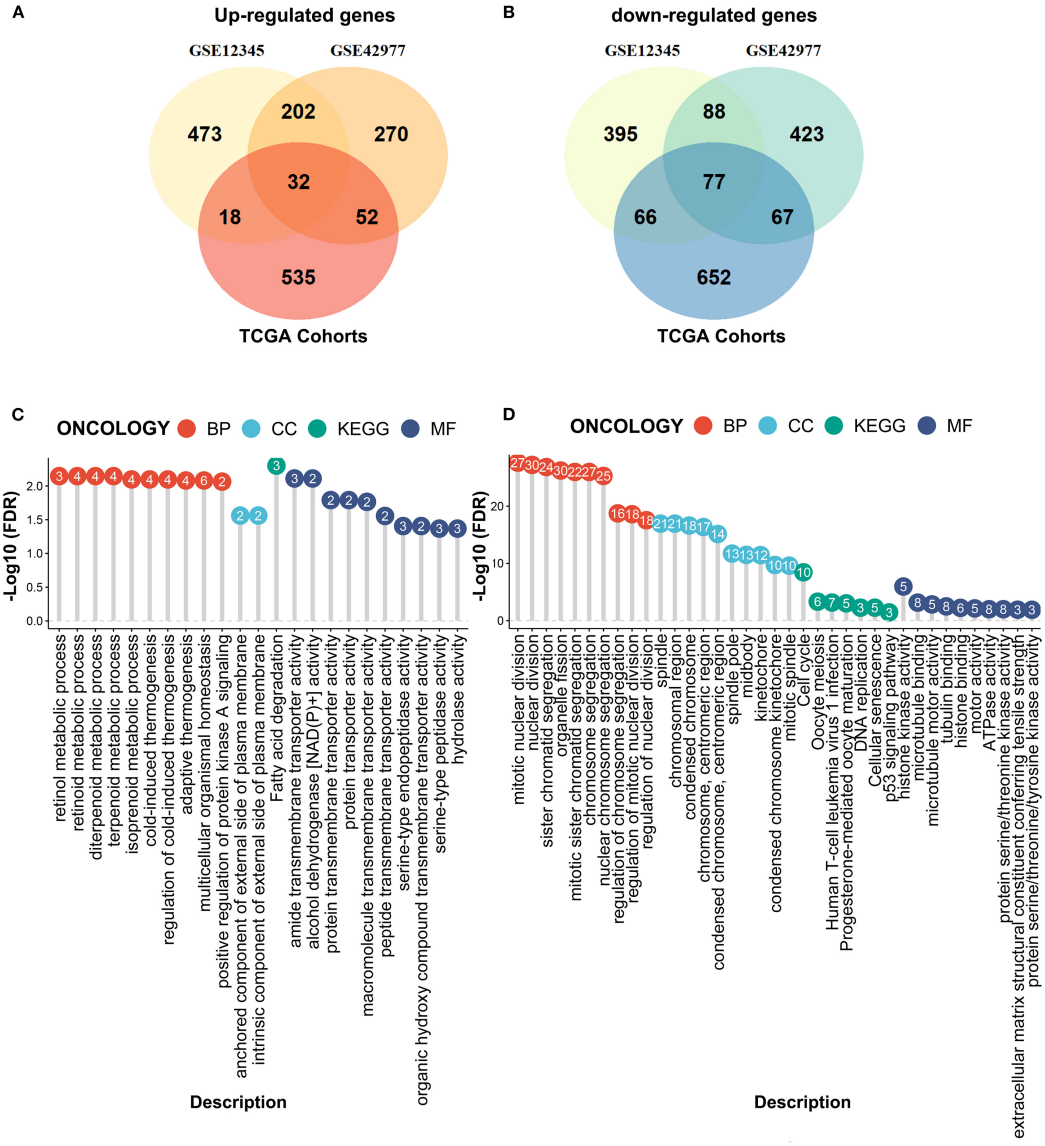
Compared the DEmRNAs obtained from the RNA-seq data and the microarray data. **(A)** The overlapped upregulated genes. **(B)** The overlapped downregulated genes. **(C)** The enrichment analyses of the overlapped upregulated genes. **(D)** The enrichment analyses of the overlapped downregulated genes.

We next performed the differential expression analyses to explore the DEGs between the MPM tissues and the normal tissues by the limma algorithm based on the microarray data. A total of 1351 DEGs (725 upregulated genes and 626 downregulated genes) were obtained from the GSE12345 dataset, and 1211 DEGs (556 upregulated genes and 655 downregulated genes) were obtained from the GSE42977 dataset (Figures 3A,B). To screen the DEGs associated with the development of the MPM, we further compared the DEGs related to the overall survival from the RNA-Seq dataset with the DEGs related to MPM obtained from the two microarray datasets, which showed the 32 overlapped upregulated DEGs and the 77 downregulated DEGs (Figures 3A,B). Similar to the above results, the enrichment analyses of the overlapped downregulated DEGs also revealed the abnormal roles of the cell division in the development of the MPM (Figure 3C). Moreover, the overlapped upregulated DEGs were mainly involved in the regulation of the metabolic processes (Figure 3D).

### Construction of an lncRNA–miRNA–mRNA ceRNA Network

**Figure 4:**
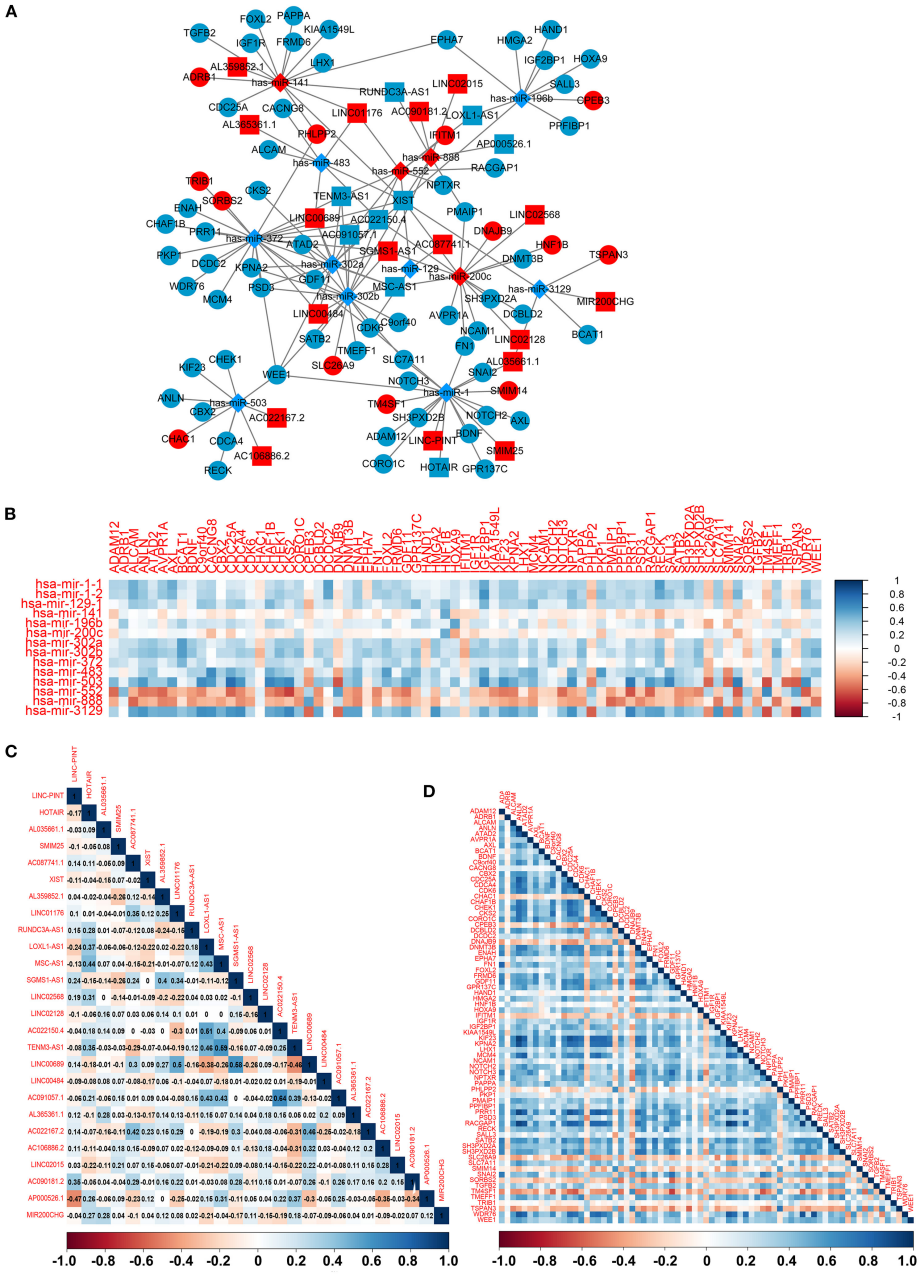
The construction of the ceRNA network among the 26 lncRNAs, 13 miRNAs, and 73 mRNAs. **(A)** The ceRNA network among lncRNA–miRNA–mRNA. Ellipse represents mRNA. Rectangle represents lncRNA. Diamond represents miRNA. Red represents upregulation, and blue represents downregulation. **(B–D)** The Pearson correlation analyses among the miRNA–mRNA, 26 lncRNAs, and 73 mRNAs.

To construct the credible ceRNA network among the lncRNAs–miRNAs–mRNAs, we input the 29 DEmiRNAs and the 358 DElncRNAs to get the interaction data between miRNAs and lncRNAs from the starBase v3.0 database. We next performed target prediction for miRNAs–mRNAs among the 29 miRNAs and the 109 overlapped mRNAs in the three databases (miRTarBase, miRDB, and TargetScan) and cross-compared the results to retain the miRNA–mRNA interactions that are consistent in all three databases. As a result, 26 lncRNAs, 13 miRNAs, and 73 mRNAs were predicted to have close interaction (Figure 4A, Table 2). To further explore the reliability of the ceRNA network, the Pearson correlations among the miRNAs–mRNAs, lncRNAs, and mRNAs were calculated based on the 80 expression profiles. The interacted miRNAs–mRNAs almost have relative high correlations with |r| > 0.4 (Figure 4B). Similar to the scattered distributions of the lncRNAs in the ceRNA network, the correlations among the lncRNAs were relatively weak, which suggested the accuracy of our ceRNA network (Figure 4C). Moreover, the majority of the mRNAs in the ceRNA network have positive correlations; the same with that many of the mRNAs in the ceRNA network are downregulated genes (Figure 4D). These results suggested the validity of the prognostic ceRNA network.

**Table 2:** The concrete interactions among the lncRNAs, miRNAs, and mRNAs in the ceRNA network.

**miRNA**	**lncRNA**	**mRNA**
hsa-miR-1	LINC-PINT, HOTAIR, AL035661.1, SMIM25, AC087741.1	NOTCH2, FN1, TM4SF1, SMIM14, SLC7A11, SH3PXD2B, CDK6, SNAI2, ADAM12, WEE1, BDNF, CORO1C, GPR137C, NOTCH3, AXL
hsa-miR-129	AC087741.1, XIST	CDK6
hsa-miR-141	AL359852.1, LINC01176, RUNDC3A-AS1, XIST	TGFB2, CDC25A, FOXL2, EPHA7, PAPPA, ADRB1, KIAA1549L, FRMD6, IGF1R, PHLPP2, LHX1
hsa-miR-196b	LOXL1-AS1, XIST	HAND1, EPHA7, HOXA9, CPEB3, PPFIBP1, HMGA2, IGF2BP1, SALL3
hsa-miR-200c	MSC-AS1, SGMS1-AS1, LINC02568, LINC02128, AC022150.4, XIST	FN1, DCBLD2, DNAJB9, SH3PXD2A, NCAM1, AVPR1A, HNF1B, PMAIP1, DNMT3B
hsa-miR-302a	TENM3-AS1, LINC00689, MSC-AS1, LINC00484, SGMS1-AS1, AC091057.1, AC022150.4, XIST	PSD3, ATAD2, CKS2, WEE1, GDF11, KPNA2
hsa-miR-302b	TENM3-AS1, LINC00689, MSC-AS1, LINC00484, SGMS1-AS1, AC091057.1, AC022150.4, XIST	SLC26A9, SATB2, SLC7A11, CDK6, PSD3, ATAD2, C9orf40, TMEFF1, WEE1, GDF11, KPNA2
hsa-miR-372	TENM3-AS1, LINC00689, MSC-AS1, LINC00484, SGMS1-AS1, AC091057.1, AC022150.4, XIST	PKP1, ENAH, SORBS2, DCDC2, CDK6, PSD3, MCM4, ATAD2, TRIB1, WEE1, GDF11, WDR76, PHLPP2, PRR11, KPNA2, CHAF1B
hsa-miR-483	AL365361.1, LINC01176, LINC00689, AC022150.4, XIST	ALCAM, CACNG8
hsa-miR-503	AC022167.2, AC106886.2	ANLN, RECK, WEE1, CHEK1, CDCA4, CHAC1, KIF23, CBX2
hsa-miR-552	TENM3-AS1, LINC01176, AC091057.1, AC022150.4, XIST, LOXL1-AS1	IFITM1, RACGAP1, NPTXR
hsa-miR-888	LINC02015, SGMS1-AS1, AC090181.2, RUNDC3A-AS1, AP000526.1, XIST	PMAIP1
hsa-miR-3129	MIR200CHG, AL035661.1, XIST	BCAT1, TSPAN3

### Construction and Evaluation of Survival Prediction Model

**Figure 5:**
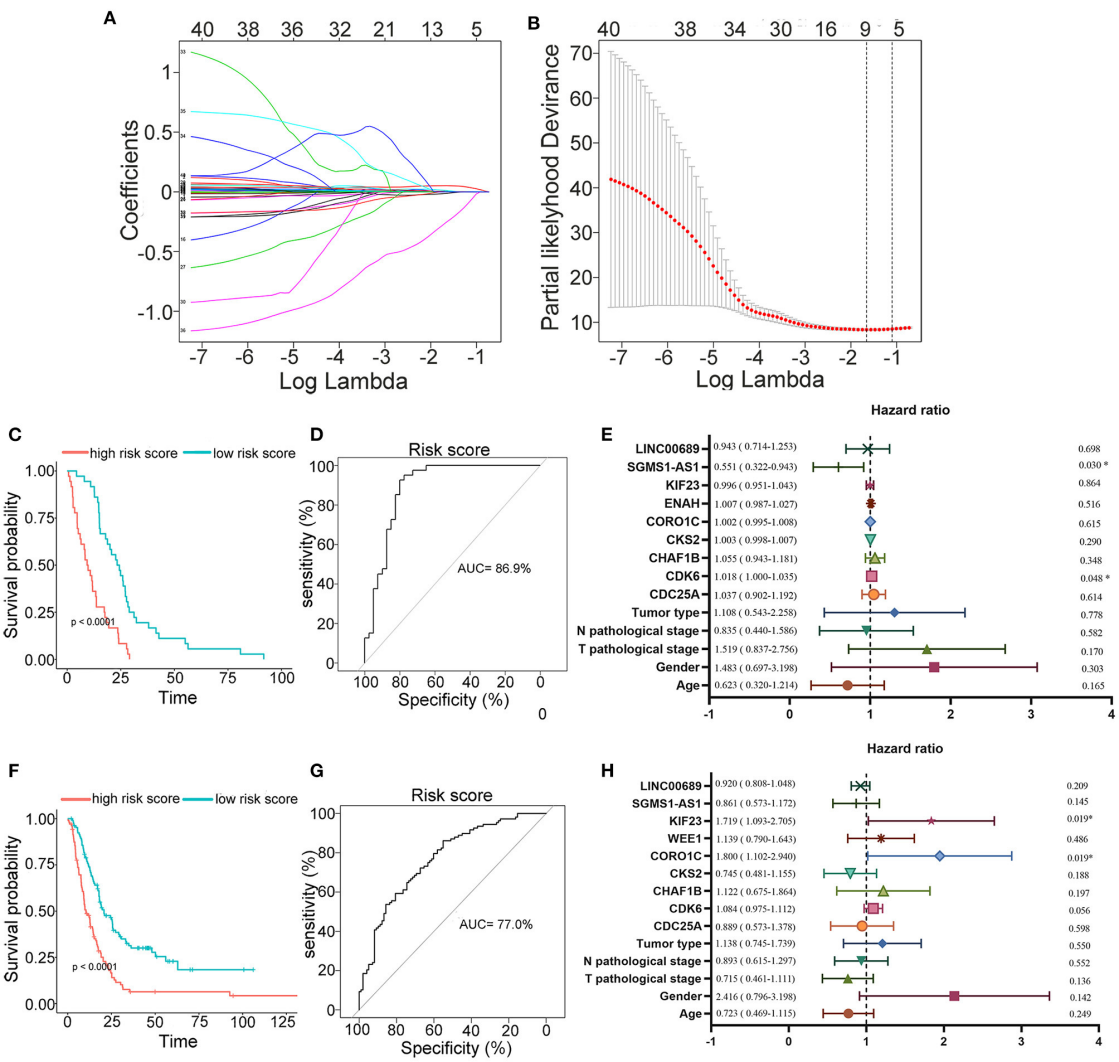
Construction, estimation, and validation of the risk model. **(A)** Least absolute shrinkage and selection operator (LASSO) coefficient profiles of the 40 genes. The value was chosen by 10-fold cross-validation. **(B)** The numbers above the graph represent the number of genes involved in the LASSO model. **(C,D)** Survival curve and ROC curve of the risk model according to the dataset of EGA. **(E)** Forest plot showing associations between the selected nine genes, the reported clinical factors, and the overall survival in the model. **(F–H)** Validation of the risk model based on the survival curve, the ROC curve, and the multivariate Cox analysis according to the EGA dataset. The significances of the survival curves were calculated by log-rank test. Also, the AUC values of ROC curves > 70% were regarded as efficient models.

**Figure 6:**
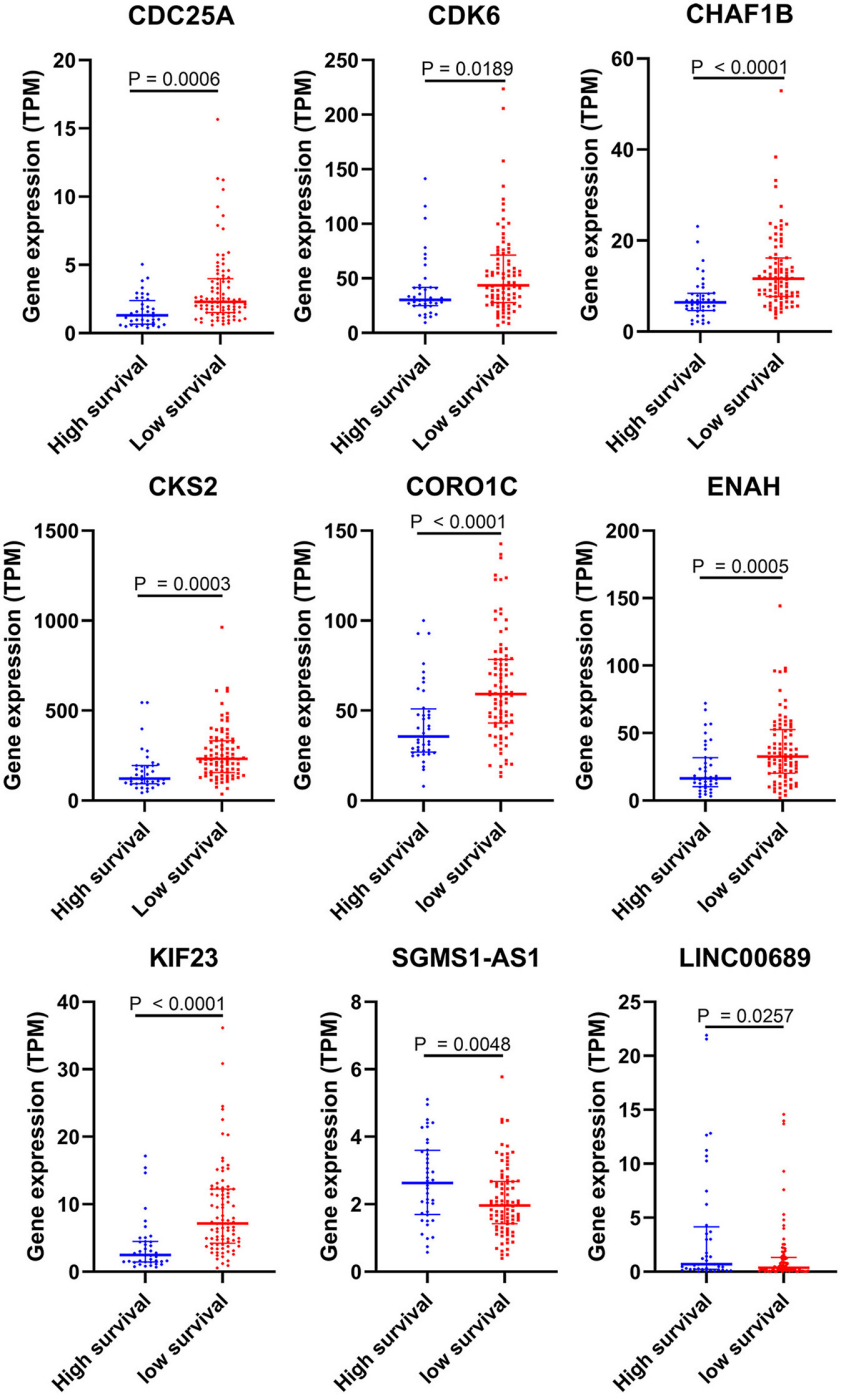
Validation of DEGs in the risk model based on the EGA MPM dataset. The statistical analyses were performed by Students' *t-*test with or without Welch correction according to the TPM of genes.

**Figure 7:**
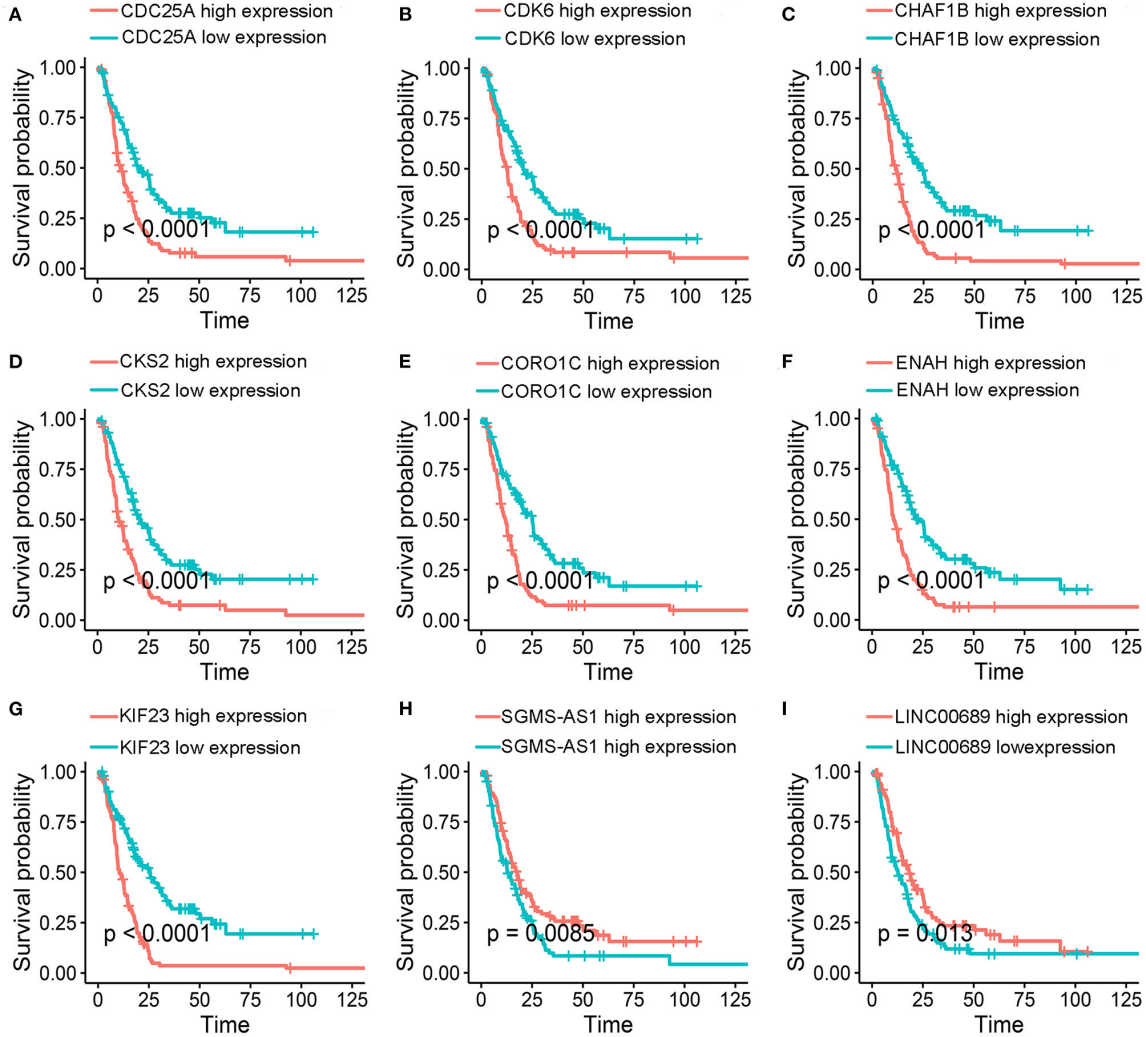
Kaplan–Meier survival analyses. **(A-I)** Validation of of the nine genes in the model based on the EGA MPM dataset. The statistical significances were determined by the log-rank test.

To further determine the association between the expression levels of the 73 mRNAs, 13 miRNAs, and 26 DElncRNAs in the ceRNA network and the overall survival of MPM patients, we performed the univariate Cox regression analyses in the 80 patients from TCGA (Table 3). Next, 52 mRNAs, six miRNAs, and 12 lncRNA were analyzed by the Kaplan–Meier survival analyses according to the grouping by the median value of expression quantities. Thirty-three mRNAs, three miRNAs, and seven lncRNAs were significant in the Kaplan–Meier survival analyses, and the significant mRNAs and lncRNAs were further screened to avoid overfitting by lasso Cox regression analyses (Figure 5A). As a result, CDC25A, CDK6, CHAF1B, CKS2, CORO1C, WEE1, KIF23, SGMS1-AS1, and LINC00689 were enrolled into the survival model (Figure 5B, Table 3). Interestingly, WEE1 and KIF23 were also identified as hub genes in the co-expressed PPI network, highlighting the importance of them related to the prognosis and the progression of MPM and the reliability of the model. Besides, the lncRNAs, WEE1, CKS2, and KIF23 included in the model were interacted with the miRNAs associated with survival time, suggesting that the model possessed high efficiency by integrating the information of mRNAs, lncRNAs, and miRNAs. Next, several clinical factors related to prognosis were also enrolled into the model, including age (≤60 or > 60), gender (female, male), T pathological stage (T1 + T2 or T3 + T4), N pathological stage (N0 + N1 or N2 + N3), and tumor type (epithelioid mesothelioma or non-epithelioid mesothelioma). According to the result of multivariate analysis, a risk model was constructed: Risk score = −0.472^*^age + 0.400^*^gender + 0.418^*^(T pathological stage)−0.180^*^(N pathological stage) + 0.102^*^ (tumor type) + 0.036^*^ CDC25A(exp) + 0.017^*^CDK6(exp) + 0.054^*^ CHAF1B(exp) + 0.003^*^ CKS2(exp) + 0.002^*^ CORO1C(exp) + 0.0237^*^ WEE1(exp)−0.004^*^ KIF23(exp)−0.0597^*^ SGMS1-AS1(exp)−0.056^*^ LINC00689(exp). The cutoff value was determined by the sensitivity equal to the specificity method from the OptimalCutpoints package, which showed the best sensitivity and specificity for prognostic prediction (AUC values = 86.9%, Figure 5D). The Kaplan–Meier survival curves showed that the overall survival of patients with high-risk scores (*n* = 32) were significantly lower than those with the low-risk scores in the three models (p < 0.001, Figure 5C). The results showed that the model is of value for prognostic prediction (Figures 5C,D). In this risk model, CDK6 and SGMS1-AS1 were significant as potential independent prognostic factors (Figure 5E). Interestingly, the histological types of MPM were associated with the risk score after the statistical analyses between the clinical traits and the risk score, similar to the previous report about the correlation between the histological types and the overall survival (Table 4). Furthermore, another dataset of MPM patients was used to validate our results. According to the TPM values of 128 patients in the EGA database, the expression of the nine DEGs in our risk model were further confirmed to be significant based on Student's *t*-test with or without Welch correction (low risk: 40 patients, high risk: 88 patients, Figure 6). Moreover, the Kaplan–Meier survival analyses of the nine DEGs in our risk model based on the data of the 211 patients from EGA also showed that all of them are closely related to the survival time of the MPM patients (Figure 7). Likewise, the Kaplan–Meier survival analyses and the ROC curves showed that the risk model has great values to estimate the prognosis of MPM (*P* < 0.001, AUC values = 77.0%, Figures 5F,G). Also, the multivariate Cox analysis was performed to verify the identified potential independent prognostic factors according to the variates from the survival model in the EGA data, which showed that CDK6 had marginal significance and KIF23 and CORO1C were significant in the model (Figure 5H). In summary, all of these results using the EGA data validated the effectiveness of the risk model.

**Table 3:** Statistical analyses associated with the OS of the mRNAs and lncRNAs in the ceRNA network.

**Gene symbol**	**Univariate analysis**			**(Kaplan–Meier survival curves, log-rank test)**
	**HR**	**CI95**	***P-*****value**	***P-*****value**
KPNA	1.02	1.01–1.02	<0.0001	3.53e-10
PRR11	1.25	1.16–1.34	<0.0001	4.82e-08
**CDC25A**	2.14	1.68–2.73	<0.0001	9.28e-08
**KIF23**	1.19	1.12–1.23	<0.0001	1.92e-07
ANLN	1.06	1.04–1.09	<0.0001	6.64e-07
**CHAF1B**	1075	1.46–2.1	<0.0001	1.20e-06
RACGAP1	1.21	1.14–1.29	<0.0001	4.29e-06
CHEK1	1.20	1.1–1.31	0.00001	4.30e-06
SATB2	1.58	1.19–2.11	<0.0019	4.35e-06
DNMT3B	3.01	1.85–4.49	<0.0001	5.10e-06
MCM	1.11	1.06–1.16	<0.0001	8.06e-06
**CORO1C**	10.4	1.03–1.05	<0.0001	2.21e-05
AXL	1.01	1.01–1.02	<0.0001	2.48e-05
CDCA4	1.14	1.08–1.2	<0.0001	3.35e-05
TMEFF1	129.56	4.61–3643.52	0.043	4.54e-05
**WEE1**	1.17	1.07–1.28	0.0006	5.15e-05
**CKS2**	1.03	1.02–1.04	<0.0001	7.01e-05
**CDK6**	1.13	1.08–1.18	<0.0001	9.47e-05
C9orf40	1.78	1.46–2.16	<0.0001	0.000147
ENAH	1.11	1.06–1.16	<0.0001	0.000354
GDF11	1.64	1.26–2.12	<0.0001	0.000413
WDR76	1.45	1.24–1.71	<0.0001	0.001005
ATAD2	1.26	1.14–1.39	<0.0001	0.00202
CPEB3	0.50	0.34–0.74	<0.0001	0.002249
NPTXR	1.25	1.12–1.39	<0.0001	0.002846
PAPPA	1.19	1.08–1.3	<0.0002	0.004774
SNAI2	1.07	1.04–1.1	<0.0001	0.006123
DCDC2	1.12	1.05–1.2	0.0014	0.006189
DNAJB9	0.98	0.96–0.99	0.0027	0.006755
AC026470.1	0.75	0.69–0.88	0.0001	0.008526
DCBLD2	1.03	1.01–1.05	0.0007	0.01441
FRMD6	1.10	1.05–1.15	0.0001	0.035087
SMIM14	0.93	0.9–0.97	0.0007	0.041198
hsa-miR-302a	1.02	1–1.04	0.0118	0.000125
hsa-miR-302b	1.01	1–1.02	0.0109	0.000134
hsa-miR-503	1.01	1–1.01	0.0062	0.007793
AC091057.1	3.92	2.21–6.97	<0.0001	0.000565
AC022150.4	3.99	2.1–7.6	<0.0001	0.000763
**SGMS1.AS1**	0.03	0–0.19	2.00E-04	3.56E-05
**LINC00689**	0.4	0.23–0.71	0.0017	0.030472
AC087741.1	0.58	0.41–0.83	0.0025	0.036343
MSC.AS1	1.26	1.08–1.48	0.0031	0.00131
RUNDC3A.AS1	10.26	1.98–53.1	0.0055	0.010718

*Bold: the genes in the survival model*.

**Table 4:** Statistical analyses between the clinical traits and the risk model.

**Subgroup**	**Low score**	**High score**	***P*-value**
**Age**			
<60	12	11	0.8049
≥60	28	29	
**Sex**			
Male	32	33	0.7745
Female	8	7	
**Tumor grade**			
STAGE I	3	6	0.4638
STAGE II+STAGE III+STAGE IV	37	34	
**Histological types**			
Biphasic mesothelioma	5	17	***0.003296***
Diffuse malignant mesothelioma (NOS)	2	3	
Epithelioid mesothelioma	33	20	

*Bold: the types of clinical traits*.

*Italic and bold: significant value*.

### Identification of Gene Modules and Functional Analyses According to WGCNA

**Figure 8:**
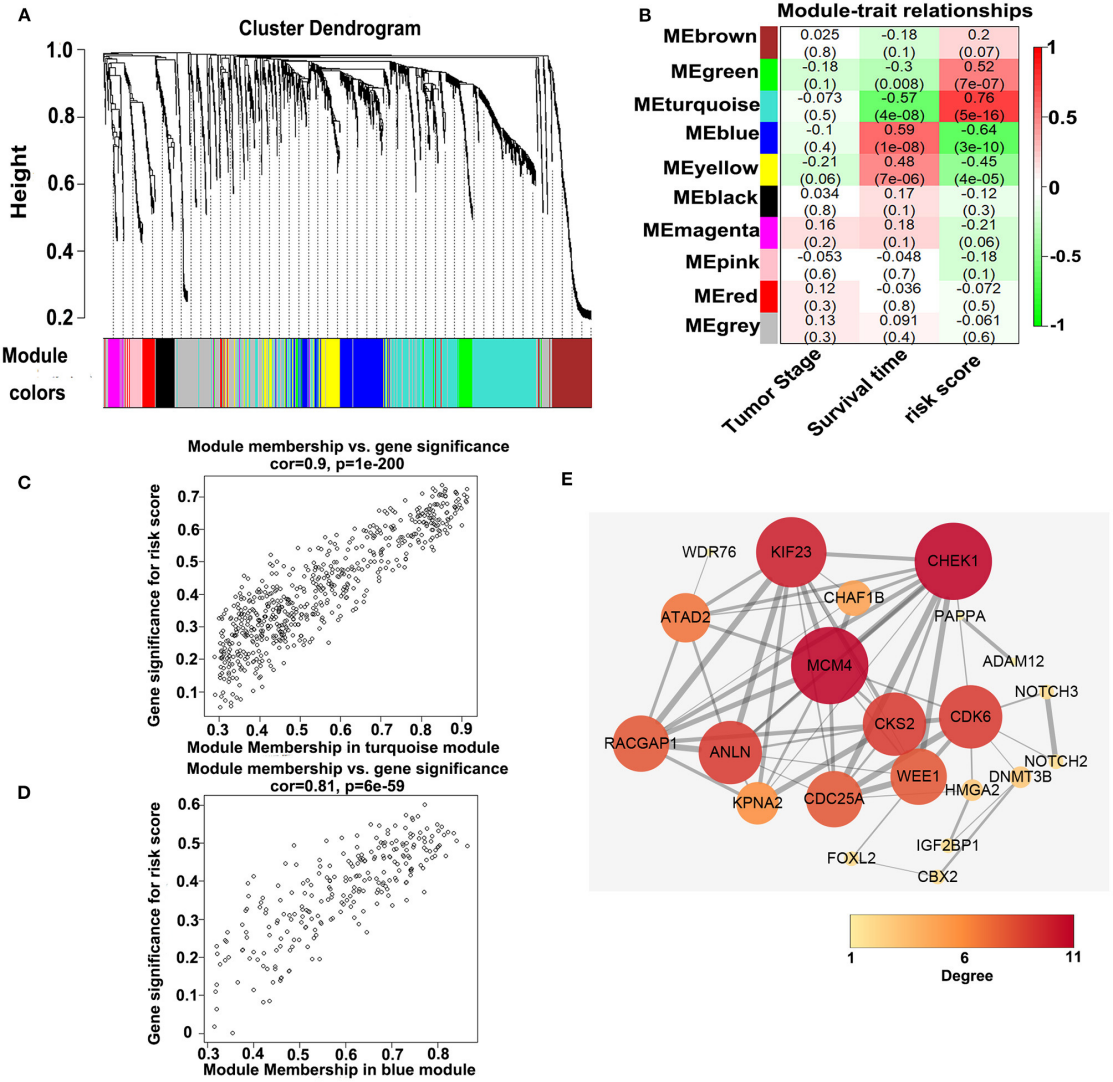
WGCNA analyses of the DEmRNAs. **(A)** Identification of the co-expression modules. **(B)** Association between the modules and the traits. **(C)** Pearson correlations between the genes in the turquoise module and the risk score. **(D)** Pearson correlations between the genes in the blue module and the risk model. **(E)** Protein–protein interaction network analysis of the overlapped mRNAs in the ceRNA network and the turquoise module. The size and color of the round represent the number of links of the protein (gene).

To investigate the potential biological function of the ceRNA network, we used the WGCNA method to cluster the DEmRNAs based on the 80 expression profiles obtained from the TCGA database. Except the non-co-expression module (gray), we identified nine co-expression modules (Figure 8A) and associated the clinical traits with the identified modules, including the tumor stage, survival time, and risk score (Figure 8B). We found that the turquoise module and the blue module were the top two most significant modules associated with the risk scores and the survival time (Figure 8B). We then performed the Pearson analyses based on the genes in the two modules and further revealed that the turquoise module was positively correlated with the risk scores and the blue module was negatively correlated with the risk scores (turquoise module—risk score: cor = 0.9, *P* = 1e-200; blue module—risk score: cor = 0.81, *P* = 6e-59; Figures 8C,D). Notably, 7 of 73 mRNAs in the ceRNA network co-expressed in the blue module and 42 of 73 mRNAs in the ceRNA network co-expressed in the turquoise module (turquoise module: 513 genes, blue module: 195 genes), which showed that the genes in the turquoise co-expression modules associated with survival were overrepresented in the ceRNA network and indicated the reliability of the ceRNA network (*P* = 2.458e-05). We then input the genes in the turquoise module into the String database to predict the PPI relationship (Figure 8E). We further analyzed the topical structure of the PPI network by 12 methods to obtain the hub genes. According to the degrees among the genes, WEE1 and KIF23 were finally selected as the hub genes in the ceRNA PPI network, suggesting that they play important roles in the MPM.

**Figure 9:**
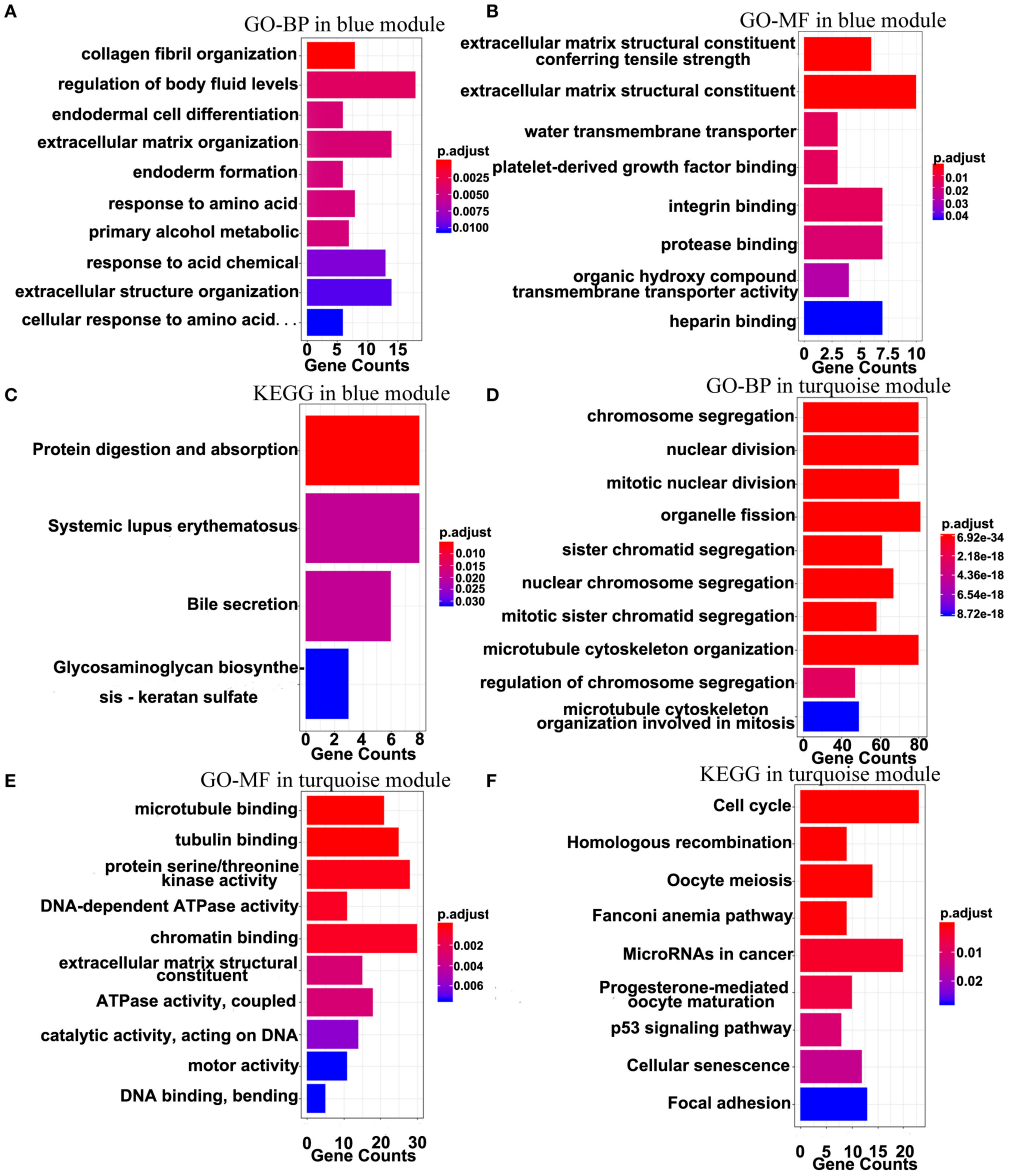
Gene-enrichment analyses of genes in the blue module and the turquoise module. **(A)** Top 10 GO-BP terms in the blue module. **(B,C)** Significant GO-MF and KEGG pathway terms in the blue module. **(D,E)** Top 10 GO-BP and GO-MF terms in the turquoise module. **(F)** Significant KEGG terms in the turquoise module. *P*-value was adjusted by the FDR method and *p* < 0.05 was significant.

The top 10 results of GO-BP, GO-MF, and KEGG pathway enrichment analyses using the genes included into the blue module and the turquoise module are shown in Figure 9. In the blue module, GO terms are mainly involved in the constituent of extracellular matrix (Figures 9A,B). As for pathways, they may be associated with the terminal symptoms of cancer patients (Figure 9C).

In the turquoise module, multiple processes related to the cell division were enriched (Figures 9D,E). Moreover, the markedly enriched pathways are associated with the cell cycle, the homologous recombination, the microRNAs in cancer, the p53 signaling pathway, etc. Figure 9F), which have been reported to participate in multiple levels of the tumorigenesis and the invasion. Overall, the results of the enrichment analyses demonstrated that the genes in the turquoise module are mainly involved in the abnormal process of cell division.

### The Immune Cell Infiltration Analysis in the ceRNA Network

**Figure 10:**
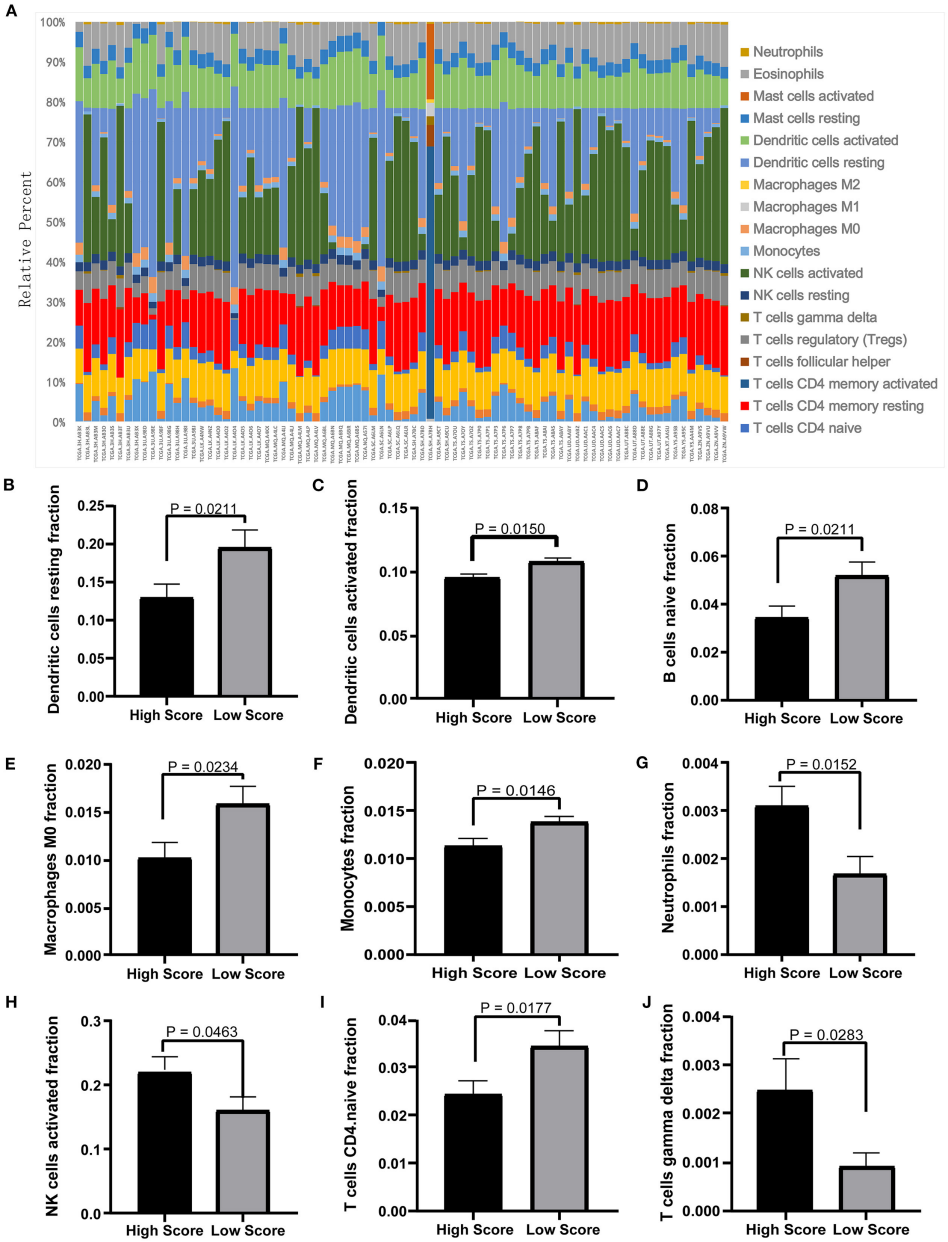
Twenty-two types of immune-cell infiltration analyses. **(A)** The overall changes of 22 types of immune cells in the MPM tissues of the 80 patients. **(B–J)** The significant differences of the immune cells between the high- and low-score groups based on the Student's *t-*test.

The tumor microenvironment involves the extracellular matrix components, the immune cells, and the other cellular components, which have partly been highlighted in multiple analyses above. To further investigate the biological function of the ceRNA network regarding the tumor microenvironment, we utilized the CIBERSORT algorithm to study the changes of tumor-infiltrating immune cells between the high- and low-risk-score groups according to the DEmRNAs in the ceRNA network (73 mRNA). We calculated the variations of the 22 types of immune cells among the 80 patients (Figure 10A) and found that the components of the nine immune cell subtypes exhibited obvious variations between the high- and low-risk groups. Compared to the low-risk group, the high-risk group possesses a higher proportion for the neutrophils, the NK cells activated, and the T cell gamma delta, while the dendritic cell resting, the dendritic cells activated, the naïve B cells, the macrophages M0, the monocytes, and the CD4-naive T cells decreased (Figures 10B–J). These results suggested that the ceRNA network may be involved in the regulation of the tumor-related microenvironment partly by changing the immune infiltration, which is potentially important for the subsequent targeted intervention.

## Discussion

MPM is a highly aggressive cancer. In the past years, great progress has been made in the knowledge of the occurrence and the development of MPM, and some target molecules and pathways were identified, including BAP1 and YAP/TAZ/TEAD oncogenic axis (21, 22). However, the pathomechanism of MPM is still largely unknown, and the clinical outcomes are highly heterogeneous. Thus, the pathogenesis of MPM requires further study, and more effective biomarkers and predictive models need to be established to satisfy the clinical setting.

Up to now, the studies aimed at screening the potential diagnostic and therapeutic targets of MPM mainly focused on the protein-coding genes (23). However, the biological processes of MPM are very complicated, which are involved in the complex interactions of the multiple types of components rather than regulation of individual molecules. Thus, it is necessary to understand the mechanism of tumor progression based on the regulatory networks. In this study, we comprehensively analyzed the expression and function of aberrant RNAs and established a ceRNA network of MPM to screen the prognostic biomarkers and construct a survival prediction model.

We firstly identified the DEGs closely related to overall survival and then annotated the abnormal function. We found that extracellular matrix organization, chromosome structure, and cell cycle were mainly involved in MPM progression. It has been well-established that the chromosome structure and the cell-cycle regulation are closely related to the cancer. Interestingly, recent studies also suggest that the tumor microenvironment is mainly formed by extracellular matrix organization, which plays an important role in the invasion and metastasis of tumor (24–26). As tumor cells proliferate, the surrounding extracellular matrix dynamically interacts with resident cells to change the architecture of the microenvironment (27). Our findings supported the importance of the extracellular matrix in MPM. In addition, the GSEA analyses based on the expressional changes of all genes also suggested anomalous morphogenesis and differentiation of epithelium and the invasive process of breast cancer partly shared the processed with the MPM development. The pathways related to oncogenes TCF21 and SMARCA2 were also enriched in the GSEA analyses. TCF21 and SMARCA2 have been reported to participate in the multiple steps of the invasion of several cancers, such as proliferation, chemoresistance, and migration (28–30), suggesting the convergence of targets among the different cancers. Besides, the overlapped DEmRNAs between the RNA-seq data and the microarray data were also identified. The function of the overlapped DEmRNAs further suggested the dysfunction of the cell division in the development of MPM.

Next, multiple databases were simultaneously used to predict the interaction among the lncRNA–miRNA-overlapped mRNAs. Finally, 26 lncRNAs, 13 miRNAs, and 73 target mRNAs were included to construct the ceRNA network. Thirty-three mRNAs, three miRNAs, and seven lncRNAs are finally identified as the genes associated with overall survival by univariate Cox regression analyses and Kaplan–Meier survival analyses. Seven mRNAs and two lncRNAs were finally selected and further used to establish survival predictive models (mRNAs: CDC25A, CDK6, CHAF1B, CKS2, CORO1C, WEE1, and KIF23; lncRNAs: SGMS1-AS1 and LINC00689). Besides, after reviews of these genes in the model, we found that all of the seven genes have been associated with multiple processes of tumor, where CDC25A, CDK6, and KIF23 have been reported to directly participate in the pathophysiological process of MPM (31–33). The expression of CDC25A has been reported to correlate with lymph-node spread of MPM (33). CDK6, WEE1, and KIF23 are thought as potential therapeutic targets, which are closely related to the prognosis of the MPM patients (31, 34). Similarly, it is noteworthy that CDK6 is also significant or marginal significant in the multivariate Cox analysis of the model according to both TCGA and EGA data, which indicated that CDK6 may be an important target and an independent prognostic factor. Although the other four genes have not been directly linked to MPM, they are widely involved in the development of cancers. CHAF1B plays a considerable role in leukemia pathogenesis and proliferation of the lung cancer and the prognosis of some cancers (32, 33). CKS2 and CORO1C participate in the growth, invasion, and prognosis of several types of cancers (35–38). Therefore, it is possible that they are also involved in the progression of MPM. LINC00689 and SGMS1-AS1 have not been extensively studied. However, there are some hints that they may be genuine tumor factors. It has been reported that LINC00689 can promote the growth, metastasis, and glycolysis of the glioma cells by completing with miR-338-3p (39). The abnormal expression of SGMS1 has been reported to regulate the epithelial-to-mesenchymal transition in several tumors (40–42). Moreover, SGMS1-AS1 may be an independent prognostic factor, which needs more data to verify. Thus, the two lncRNAs are likely to participate in the invasive process of MPM. The functions of all these genes are worth further studying, especially the concrete validation of the lncRNA–miRNA–mRNA axis. The genes included in the model exhibited high efficiency by integrating the information of mRNAs, lncRNAs, and miRNAs. The other genes may also have high clinical significance. Interestingly, some of these genes out of the models were also directly associated with the MPM, which were mainly regarded as potential therapeutic targets, including CHEK1, DNMT3B, AXL, PAPPA, and miR-302b. Previous studies suggested that these genes are involved in the multiple antineoplastic processes of MPM, such as relief of chemo- and radioresistance, anti-proliferation, inhibition of growth and migration, and induction of apoptosis (31, 43–48). Only two small-molecule drugs aiming to target the identified genes have been preliminarily evaluated, including palbociclib targeting CDK6 and AZD1775 targeting WEE1. Therefore, more preclinical experiments should be conducted to screen the drugs targeting the corresponding genes and assess the antitumor effect of these identified targets before the clinical studies, especially for these identified lncRNAs, in consideration of their central roles in the ceRNA network.

We next clustered the nine co-expression modules from the 1500 DEmRNAs based on the method of WGCNA and found that the blue module and the turquoise module were significantly associated with the overall survival and the risk model. Importantly, the two hub genes WEE1 and KIF23 in the turquoise co-expression PPI network have been previously reported to play important roles in the aggressive property of MPM, whose inhibition was previously regarded as the promising therapeutic targets (49, 50). Interestingly, WEE1 and KIF23 are also included into the survival model, which indicated the validity of the model and the ceRNA network. Besides, KIF23 was identified as an independent survival predictor in EGA data, but not in the TCGA data, possibly due to insufficient data. As for the biological functions of KIF23 and WEE1 in MPM, they remain largely unknown. The ceRNA mechanisms of the two genes in MPM also have not been assessed, which may be important for the growth and metastasis of the MPM cells. Similarly, the gene enrichment analyses further suggested that the blue module mainly regulates the function of the extracellular matrix, indicating that the ceRNA network could affect the tumor microenvironment and consequently contribute to MPM progression. Moreover, the functions of the turquoise module were mainly involved in the process of cell division and in the structure of cells, whose dysfunction is widely regarded as the initiation and the invasive factor of cancer. In other words, we deduce that the ceRNA network mainly regulates the invasion of MPM by regulating the extracellular matrix and the cell division.

The tumor-related microenvironment also includes the immune cells, the fibroblasts, and the endothelial cells, which could inhibit the development of tumor. However, with the progression of tumor, the inhibitory signals and the immune cells could be circumvented by changing the tumor microenvironment (51). Therefore, there is a complex relationship between the tumor cells and the immune cells, which plays important roles in either elimination of tumor cells or invasion of rumors. Thus, we calculated the proportions of the 22 types of immune cells by the deconvolution of the DEmRNAs from the ceRNA network, whose result suggested that the ceRNA network may also be involved in the infiltration of multiple immune cells and the regulation of the microenvironment of MPM.

To conclude, we have identified a number of potential prognostic genes and analyzed their functions related to the progression of the MPM. A ceRNA regulatory network was accordingly constructed, where 33 mRNAs, three miRNAs, and seven lncRNAs are finally identified as the genes associated with the overall survival. Then an efficient risk score assessment system based on two lncRNAs, seven mRNAs, and five clinical factors were accordingly established and validated to predict the overall survival of MPM patients by two independent MPM case–control studies. Besides, the influence of the ceRNA network on the tumor microenvironment has also been assessed from several perspectives, providing a further understanding of the mechanisms of MPM progression. The follow-up clinical and experimental researches are needed to further optimize the models and concretely clarify the role that these genes play in the MPM.

## Data Availability Statement

The original contributions presented in the study are included in the article/supplementary materials, further inquiries can be directed to the corresponding author/s.

## Ethics Statement

Ethical approval was not provided for this study on human participants because the human datasets were obtained from the public database including TCGA, EGA and GEO, which have been approved by the ethical reviews. The patients/participants provided their written informed consent to participate in this study.

## Author Contributions

WD, BX, and PH: conceptualization. WD and KW: methodology, software, and writing—original draft preparation. WD, YD, and XYC: validation. WD and XFC: formal analysis. BX and PH: writing—review and editing, supervision, and project administration. WD: visualization. BX: funding acquisition. All authors have read and agreed to the published version of the manuscript.

## Conflict of Interest

The authors declare that the research was conducted in the absence of any commercial or financial relationships that could be construed as a potential conflict of interest.
